# Supplementary material for: Potential Effect of Pseudevernia furfuracea (L.) Zopf Extract and Metabolite Physodic Acid on Tumour Microenvironment Modulation in MCF-10A Cells
Source: Biomolecules. 2021 Mar 12;11(3):420. doi: 10.3390/biom11030420 (PMC8000760; doi:10.3390/biom11030420)
Supplement: Supplementary file 1 [file biomolecules-11-00420-s001.pdf]

biomolecules

MDPI

Article

Potential effect of *Pseudevernia furfuracea* (L.) Zopf extract and metabolite physodic acid on tumour microenvironment modulation in MCF-10A cells

Klaudia Petrova [1•], Martin Kello [1*•], Tomas Kuruc [1], Miriam Backorova [2], Eva Petrovova [3], Michal Goga [5,6] Maria Vilkova [4], Dajana Rucova[6], Martin Backor [6] and Jan Mojzis [1*]

1 Department of Pharmacology, Faculty of Medicine, Pavol Jozef Šafárik University, 040 01 Košice, Slovakia; claudy.petrova@gmail.com (K.P); tomaskuruc@gmail.com (T.K); jan.mojzis@upjs.sk (J.M); kellomartin@yahoo.com (M.K)
2 Department of Pharmaceutical Technology, Pharmacognosy and Botany, University of Veterinary Medicine and Pharmacy, 041 81, Košice, Slovakia; miriambackorova@gmail.com (Mi.B.)
3 Department of Anatomy, Histology and Physiology, University of Veterinary Medicine and Pharmacy, 041 81 Košice, Slovakia; petrovova.e@gmail.com (E.P.)
4 Department of NMR Spectroscopy, Institute of Chemistry, Faculty of Science, Pavol Jozef Šafárik University, Moyzesova 11, 040 11, Košice, Slovakia; maria.vilkova@upjs.sk (M.V.)
5 Core Facility Cell Imaging and Ultrastructure Research, University of Vienna, Althanstrasse 14, 1090, Vienna, Austria; michal.goga@univie.ac.at
6 Department of Botany, Institute of Biology and Ecology, Faculty of Science, Pavol Jozef Šafárik University, Mánesova 23, 041 67, Košice, Slovakia. michal.goga@univie.ac.at. (M.G); dajana.rucova@gmail.com (D.R); martin.backor@upjs.sk (Ma.B.)
* Correspondence: kellomartin@yahoo.com (M.K.); jan.mojzis@upjs.sk (J.M.)
• Klaudia Petrova and Martin Kello contributed equally to this work

Citation: Petrova, K.; Kello, M.; Kuruc, T.; Backorova, M.; Petrovova, E.; Goga, M.; Rucova, D.; Backor, M.; Mojzis, J. Potential effect of *Pseudevernia furfuracea* (L.) Zopf extract and metabolite physodic acid on tumour microenvironment modulation in MCF-10A cells. *Biomolecules* **2021**, *11*, 420. https://doi.org/10.3390/biom11030420

Received: 1 February 2021
Accepted: 9 March 2021
Published: 11 March 2021

Publisher's Note: MDPI stays neutral with regard to jurisdictional claims in published maps and institutional affiliations.

Copyright: © 2021 by the authors. Submitted for possible open access publication under the terms and conditions of the Creative Commons Attribution (CC BY) license (http://creativecommons.org/licenses/by/4.0/).

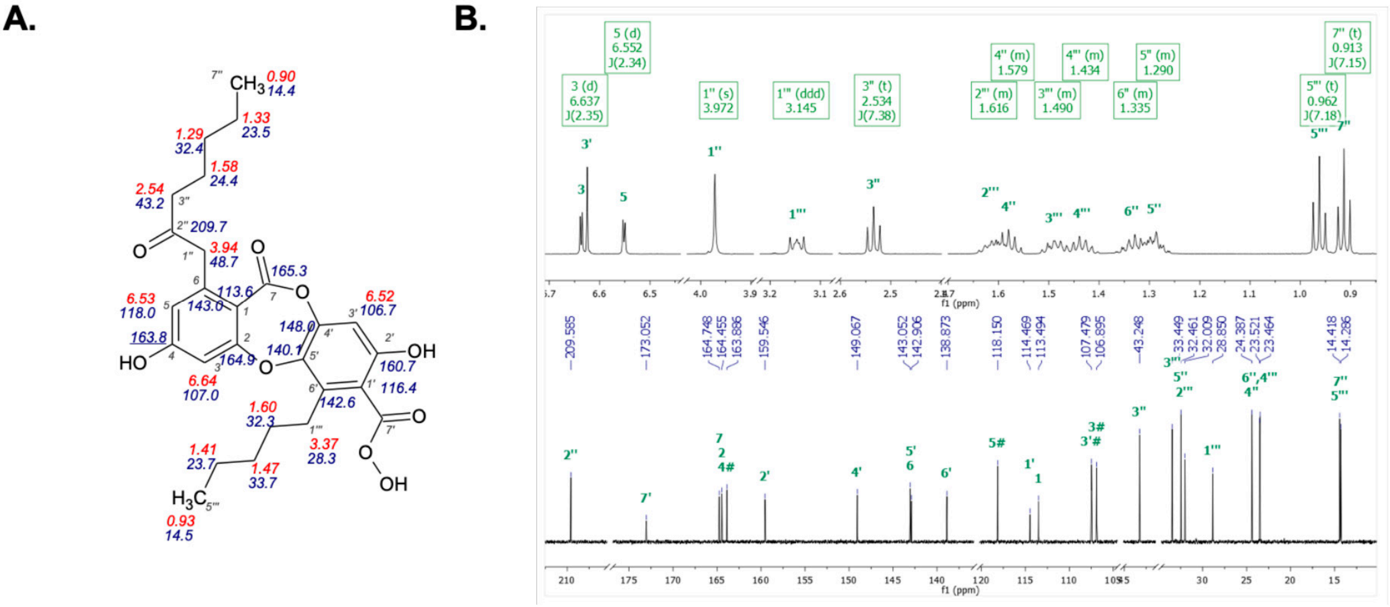

Figure S1. (**A**) Structure of physodic acid together with atom numbering and (**B**) ^{1}H (600 MHz, CD_3OD) and ^{13}C NMR (150 MHz, CD_3OD) spectrum.

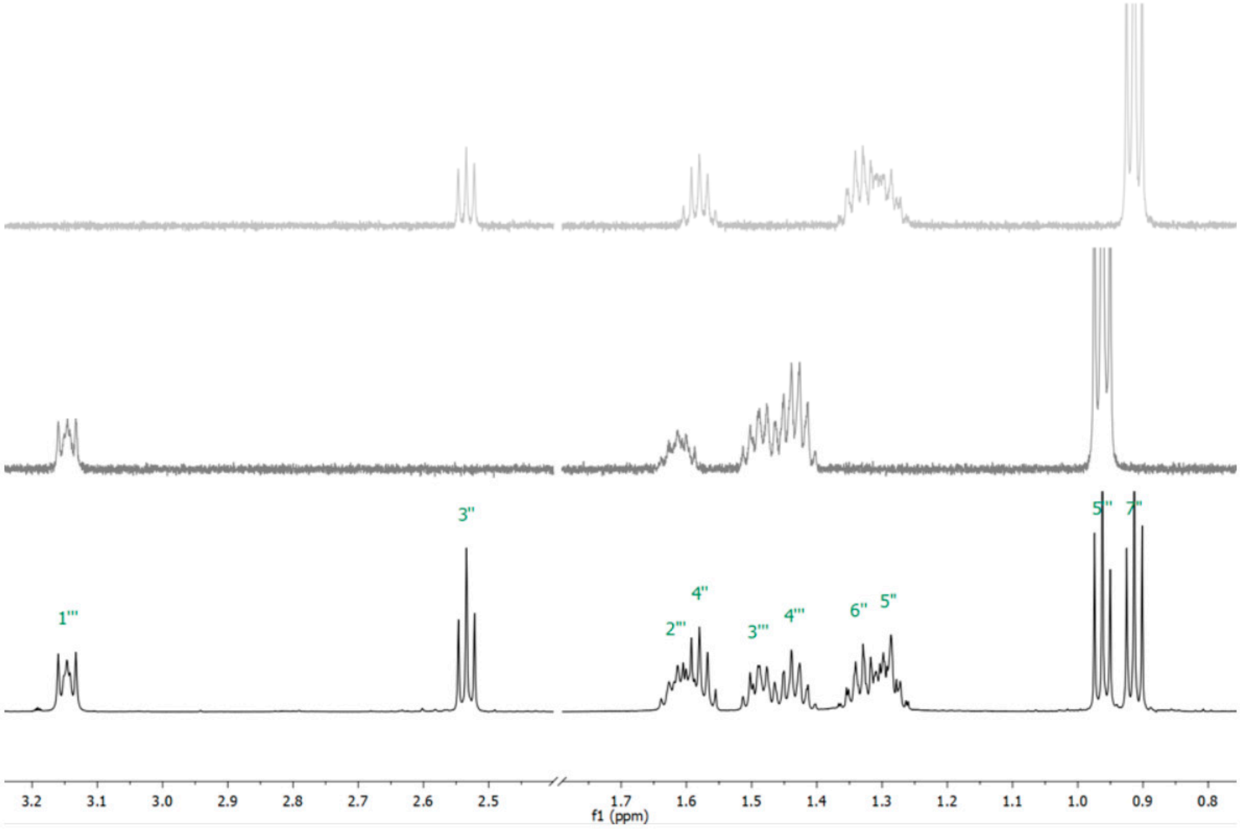

Figure S2. 1D TOCSY (600 MHz, CD_3OD) spectra of physodic acid showing only aliphatic regions.

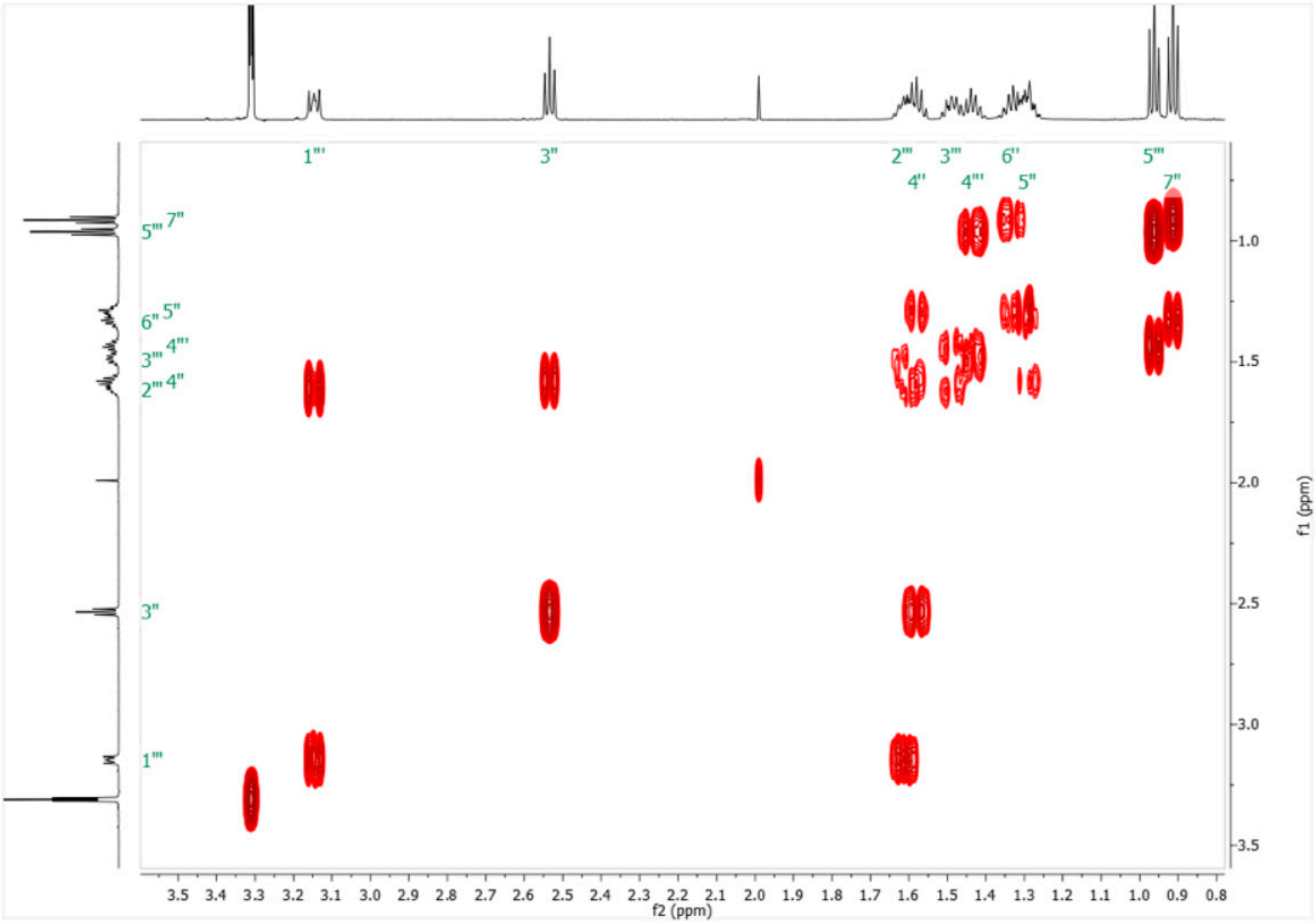

Figure S3. ^{1}H, ^{1}H-COSY spectrum (600 MHz, CD_3OD) of physodic acid showing only aliphatic region.

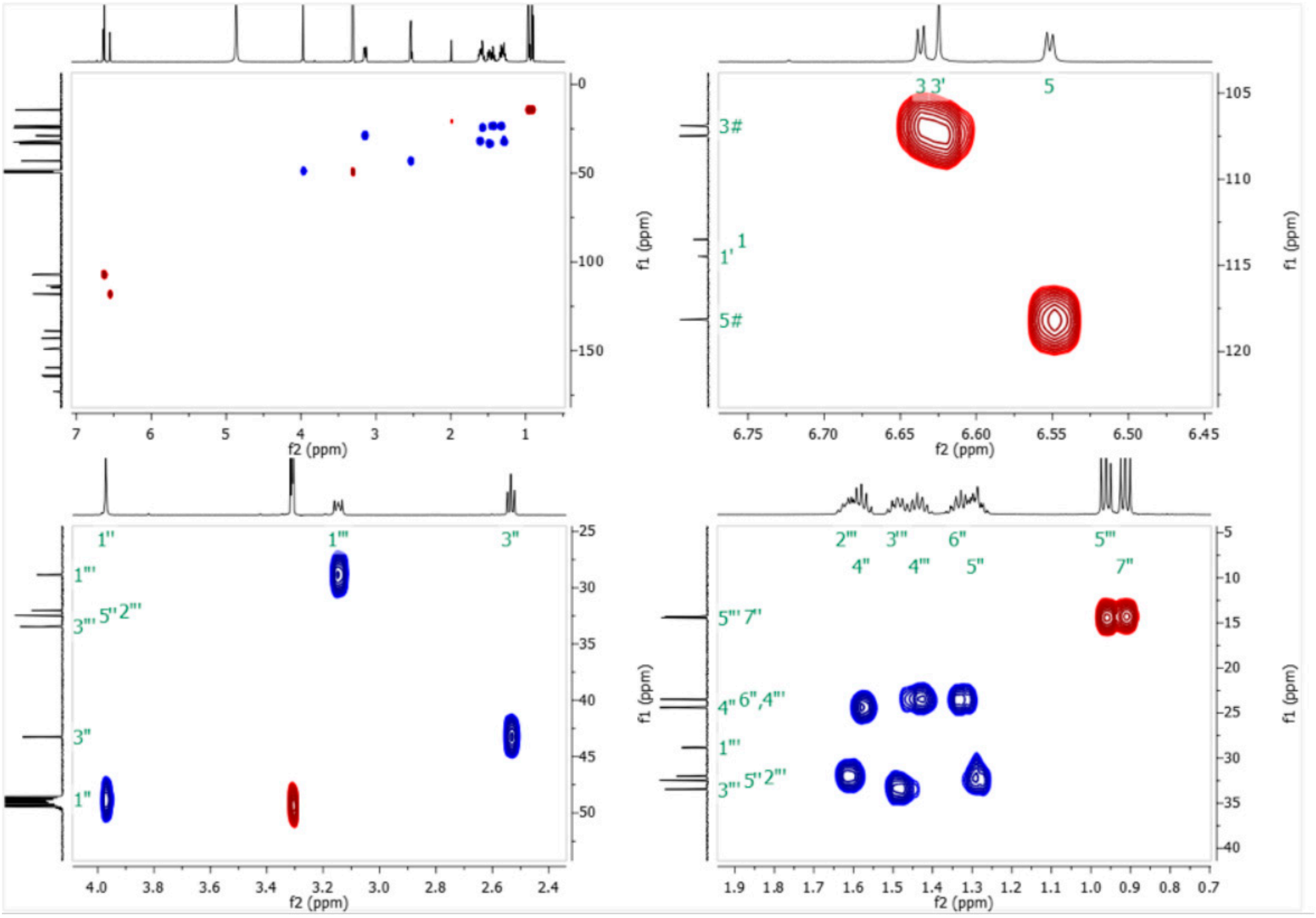

Figure S4. ^{1}H, ^{13}C-HSQC spectra (CD_3OD) of physodic acid.

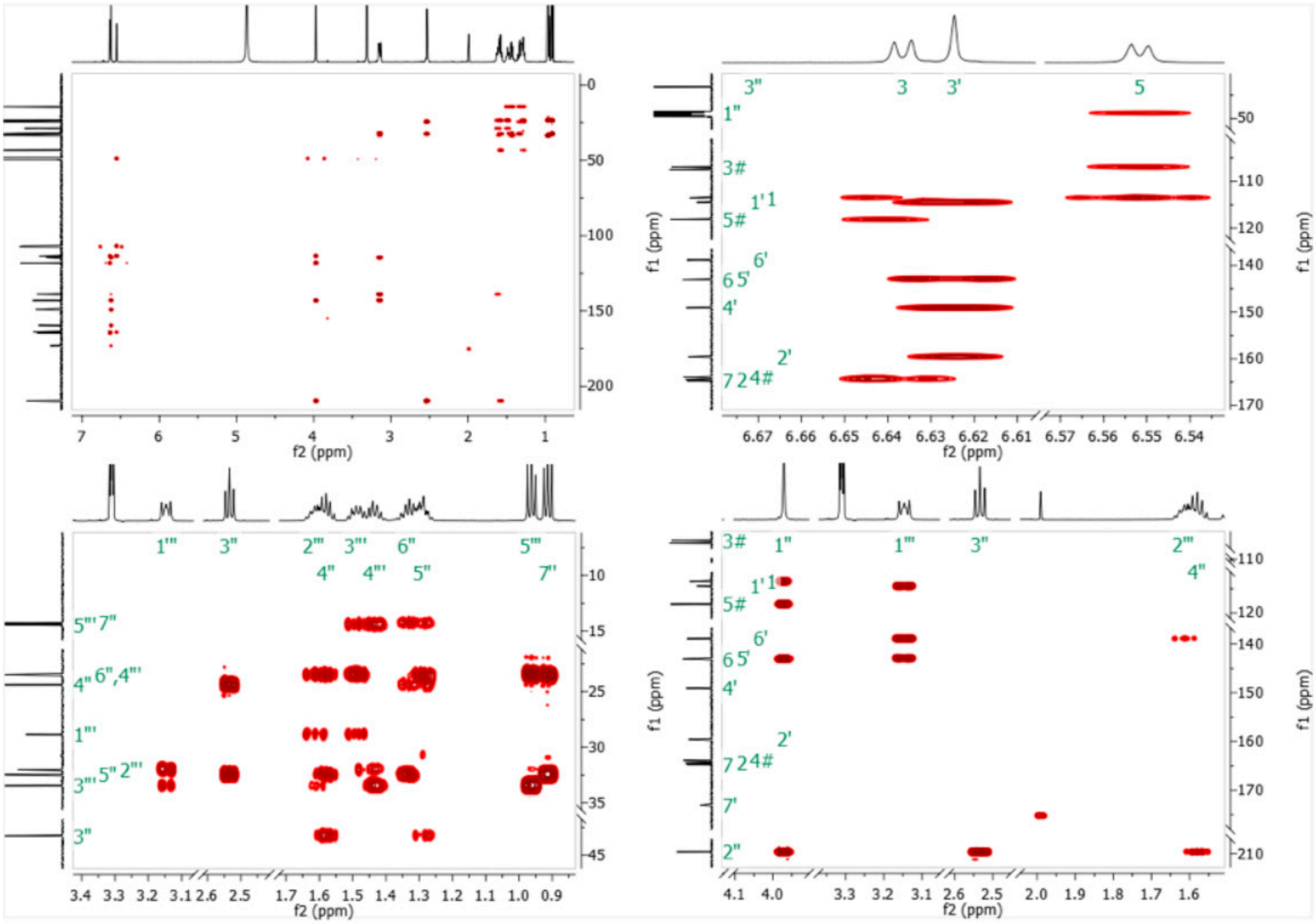

Figure S5. ^{1}H, ^{13}C-HMBC spectra (CD_3OD) of physodic acid.

Table S1. ^{1}H (600 MHz, CD_3OD) and ^{13}C (600 MHz, CD_3OD) NMR chemical shift (δ, ppm) of physodic acid.

**No.**	**δ_H (mult. *J*)**	**δ_C**
1		113.5
2		164.5
3	6.63 (d, 2.4)	106.9
4		163.9
5	6.55 (d, 2.4)	118.2
6		143.1
7		164.7
1′		114.5
2′		159.5
3′	6.63 (s)	107.5
4′		149.1
5′		142.9
6′		138.9
7′		173.1
1′′	3.97 (s)	48.8
2′′		209.6
3′′	2.53 (t, 7.4)	43.2
4′′	1.58 (m)	24.4
5′′	1.29 (m)	32.5
6′′	1.34 (m)	23.5
7′′	0.91 (t, 7.2)	14.3
1′′′	3.15 (ddd, 10.8)	28.9
2′′′	1.62 (m)	32.0
3′′′	1.49 (m)	33.4
4′′′	1.43 (m)	23.5
5′′′	0.96 (t, 7.4)	14.4
